# Adherence to evidence based care practices for childbirth before and after a quality improvement intervention in health facilities of Rajasthan, India

**DOI:** 10.1186/1471-2393-14-270

**Published:** 2014-08-13

**Authors:** Kirti Iyengar, Motilal Jain, Sunil Thomas, Kalpana Dashora, William Liu, Paramsukh Saini, Rajesh Dattatreya, Indrani Parker, Sharad Iyengar

**Affiliations:** Action Research & Training for Health, Udaipur, Rajasthan India; State Institute of Health and Family Welfare, Government of Rajasthan, Jaipur, Rajasthan India; United Nations Population Fund, Jaipur, Rajasthan India; Northwestern Feinberg School of Medicine, Chicago, Illinois USA

**Keywords:** Childbirth, Quality of care, Intrapartum, Evidence based delivery care, Rajasthan, Maternal care, Perinatal mortality, Institutional delivery

## Abstract

**Background:**

After the launch of Janani Suraksha Yojana, a conditional cash transfer scheme in India, the proportion of women giving birth in institutions has rapidly increased. However, there are important gaps in quality of childbirth services during institutional deliveries. The aim of this intervention was to improve the quality of childbirth services in selected high caseload public health facilities of 10 districts of Rajasthan. This intervention titled “Parijaat” was designed by Action Research & Training for Health, in partnership with the state government and United Nations Population Fund.

**Methods:**

The intervention was carried out in 44 public health facilities in 10 districts of Rajasthan, India. These included district hospitals (9), community health centres (32) and primary health centres (3). The main intervention was orientation training of doctors and program managers and regular visits to facilities involving assessment, feedback, training and action. The adherence to evidence based practices before, during and after this intervention were measured using structured checklists and scoring sheets. Main outcome measures included changes in practices during labour, delivery or immediate postpartum period.

**Results:**

Use of several unnecessary or harmful practices reduced significantly. Most importantly, proportion of facilities using routine augmentation of labour reduced (p = 0), episiotomy for primigravidas (p = 0.0003), fundal pressure (p = 0.0003), and routine suction of newborns (0 = 0.0005). Among the beneficial practices, use of oxytocin after delivery increased (p = 0.0001) and the practice of listening foetal heart sounds during labour (p = 0.0001). Some practices did not show any improvements, such as dorsal position for delivery, use of partograph, and hand-washing.

**Conclusions:**

An intervention based on repeated facility visits combined with actions at the level of decision makers can lead to substantial improvements in quality of childbirth practices at health facilities.

## Background

Deaths within the first few hours after childbirth contribute to a significant proportion of maternal and neonatal mortality in developing countries. Skilled attendance at birth and timely action to detect and address complications have been recognised as essential for reducing maternal and perinatal mortality [1]. To impact mortality however, the rising rate of institutional delivery worldwide must be accompanied by commensurate improvements in quality of care [2]. More specifically, with two million intrapartum related stillbirths and neonatal deaths occuring each year, improving the quality of intrapartum care has been recognised as critical to to the achievement of Millennium Development Goals (MDG) 4 and 5A [1].

After Government of India launched a conditional cash transfer scheme to provide incentives to women giving birth in public health facilities [3], there was substantial increase in the number of women giving birth in hospitals and health centres. Over 5 years, this proportion increased nationwide from 34.9% in 2006 to 60.5% in 2010, and in the northern state of Rajasthan from 24.1% in 2006 to 68.0% in 2010 [4]. However, the maternal mortality ratio (MMR) has not declined to a commensurate extent. While the proportion of women delivering in institutions almost tripled between 2001–03 and 2007–09 in Rajasthan, the maternal mortality ratio (MMR) declined 29% from 445 to 318 per 100,000 live births over roughly the same period [5, 6]. A recent analysis of data from 284 districts in nine states of India could not detect significant association between the proportion of women delivering in health facilities and MMR [7]. Similarly, another study that analysed impact of National Rural Health Mission (NRHM) interventions on perinatal mortality in 20 Indian states revealed that while hospital deliveries increased by 57% from 2005 to 2008, the relative decline in perinatal mortality rate in rural areas was only 2.5% [8]. The same analysis also reported 167% increase in institutional deliveries in the state of Rajasthan, with 4% decline in rural perinatal mortality. The Lancet series on stillbirths estimated that 56.6% of all stillbirths in South Asia are related to intrapartum causes [9]. Improving intrapartum monitoring and timely access to Caesarean section in low and middle-income countries appears to be key to reducing intrapartum stillbirths [10].

A few studies point to important gaps in the quality of institutional delivery in Rajasthan. A cross sectional survey of recently delivered women during 2007–08 reported that 85% of those delivering in institutions received injections to speed up labour, 67% were subjected to strong manual fundal pressure by birth attendants, and over half were discharged too early, less than 24 hours after delivery [11]. A qualitative study featuring observations of women in labour revealed that maternal and fetal condition (by way of blood pressure and fetal heart sounds) were not monitored adequately during labour, the practice of strong fundal pressure and routine augmentation using intramuscular or intravenous oxytocin was routine, and preparedness for neonatal resuscitation was minimal [12]. Other studies from India have also showed that labour augmentation was used in more than 70% of institutional deliveries [13, 14].

Lack of adherence to evidence based care practices during delivery has been found to be common in middle eastern countries [15] including Jordan, in which labour augmentation and lithotomy birthing positions were observed in 95% and 100% of hospital deliveries respectively [16]. An observational study in an Egyptian hospital showed that 91% of labour was augmented and that 93% of such women received inappropriate augmentation, that too without adequate monitoring [17].

A few studies point to the relationship between augmentation of labour and perinatal mortality in low income countries. Dujardin and colleagues analysed the risk of stillbirth and neonatal resuscitation associated with the use of oxytocin for three developing countries. Their results showed a relative risk of 1.9 for stillbirth [18]. A review of rates, trends, indications and risks associated with induction and augmentation in low income countries reveals high rates of use of oxytocin and misoprostol for induction and augmentation of labour and their association with stillbirth, neonatal resuscitation and uterine rupture [19]. A case control study from Uganda [20] demonstrated significant association (odds ratio 5.76 (2.20-15.05)) between augmentation of labour and birth asphyxia , while a verbal autopsy study of stillbirths and neonatal deaths in Nepal concluded that use of injections to accelerate home delivery had contributed to deaths from birth asphyxia [21]. The relationship of labour augmentation to adverse perinatal outcomes underlies the WHO recommendations that oxytocin or prostaglandins should be used to augment labour only with adequate maternal-fetal monitoring, and that too in facilities having immediate access to caesarean section and neonatal resuscitation [22, 23]. Adherence to evidence based care practices is essential not only for achieving better maternal and perinatal outcomes, but also for reducing maternal morbidity and for increasing acceptance among women. For example, routine episiotomy is linked to more frequent posterior perineal trauma and suturing [24] and this might deter women from seeking institutional delivery. Similarly, routine pubic shaving and enema during labour are unnecessary and make the experience of childbirth unpleasant for women [22, 25].

A comprehensive review of strategies has concluded that intrapartum related neonatal deaths can be substantially reduced by improving quality of care for all childbirths that occur within health facilities [26]. A range of simple, effective and affordable interventions exist, though coverage of effective interventions is low. “Parijaat”, emerged as a collaborative initiative for improving the quality of facility based delivery services as a result of consultations between representatives of the state government’s Medical, Health & Family Welfare Department, UNFPA, UNICEF and Action Research & Training for Health (ARTH), a not-for-profit organization. This paper describes the resultant intervention that was implemented in 44 high workload public health facilities of 10 districts, and its impact on key inputs and maternal-newborn care practices.

## Methods

### Design

We designed an intervention to improve the quality of childbirth practices in consultation with state and district managers of the NRHM. It was not possible to use an intervention-control design because the NRHM is mandated to reach all facilities with interventions of likely effectiveness. The intervention comprised minimum quarterly visits to all 44 selected health facilities for quality improvement, during which the team used standard checklists to closely document changes in equipment, supplies and practices. Findings from the documentation have been presented in this paper, which therefore mirrors an uncontrolled before and after health system intervention. The main outcomes were provision of selected inputs and adherence to evidence-based care practices before and after the intervention, which was phased into individual facilities between Dec 2010 and Aug 2011, and was implemented for 12–18 months in each facility.

### Building consensus and defining the intervention

Close collaboration with the state government was established from the design stage. A core group of 3 persons reviewed the published literature to identify the major gaps in childbirth practices and their prevalence. Further, they also relied on their own or their colleagues’ experiences of observations of deliveries in public health facilities of Rajasthan. Some of the key problems identified were little or no monitoring of maternal and fetal condition during labour, rampant augmentation of labour and fundal pressure, routine episiotomy, inadequate readiness to deal with birth asphyxia, lack of hand-washing for delivery and newborn care and inadequate care of the mother and newborn in the postpartum ward. The group also identified the gaps in inputs- labour rooms and labour table were often dirty, with old blood stains, blood pressure instruments, stethoscopes and neonatal ambu bags were often not readily available in the labour room.

**Figure 1:**
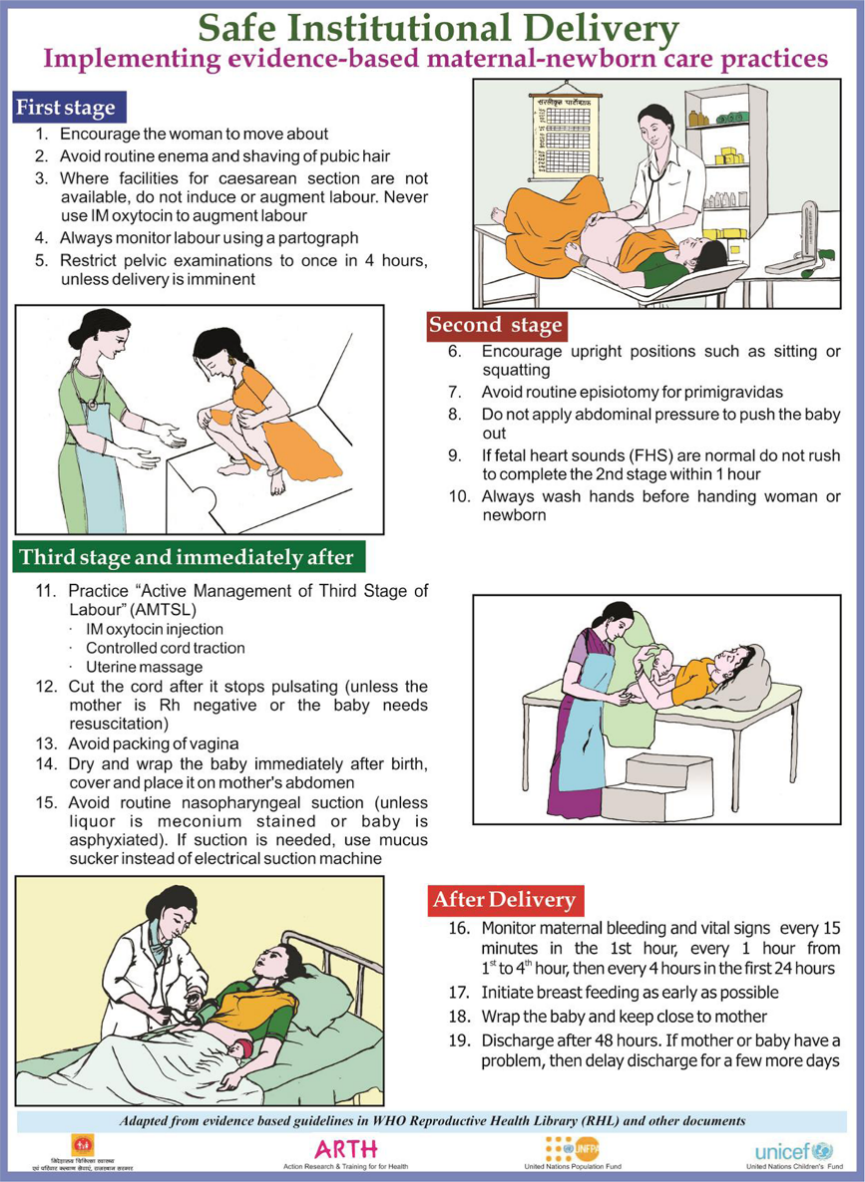
**Poster listing evidence based practices for each stage of labour. ©** Action Research & Training for Health. This material has been designed, field tested and produced by Action Research & Training for Health (ARTH) in consultation with partners who endorsed the final product and approved its publication. It may be reproduced or translated for non-commercial purposes without prior permission, provided the source is acknowledged and a copy is sent to ARTH, G1-2, Satyam, Ramgiri, Badgaon, Udaipur 313011, Rajasthan, India (email: arthindia@gmail.com).

The core group reviewed published literature (including Cochrane reviews and the WHO Reproductive Health Library [22, 27–29]), to narrow down to those evidence based care practices during each stage of labour and immediate postpartum period, that were likely to either impact mortality or morbidity, consume valuable provider time without commensurate benefit, or make the service less acceptable to women. The group then selected practices that would be amenable to ready assessment and change – for example, the group felt that companions are often present with women during labour and hence the practice did not require much change; frequency of vaginal examinations was difficult to accurately assess and was not included. Shortlisted practices were presented at an expert group meeting, 19 were finalized and later developed into a chart outlining key evidence-based care practices categorized by stage of labour (Figure 1). Further, a guidebook titled “Recommendations for key delivery and newborn care practices in health facilities of Rajasthan” was developed for clinicians. It presented the evidence for selected practices that were expected to have greater impact on maternal and perinatal outcomes.

A state level consultation brought together state health policy makers, clinical heads of obstetrics-gynecology and pediatrics from medical colleges, senior health directorate and district staff, UNFPA and UNICEF, to build consensus on strengthening evidence based care practices within district health facilities. Participants reviewed the current situation with respect to delivery and newborn care services in the state and prioritized actions to improve quality of care.

### Identification of facilities

In consultation with the government health department, facilities in which at least 900 women had delivered in the previous year (2009–10), were identified in 10 districts located in the southern and north-eastern parts of the state. Six of these districts had large populations belonging to marginalized tribal groups. In this paper we report on the experience with 44 government facilities that included 9 district hospitals, 32 community health centres (CHCs) and 3 primary health centres (PHCs).

### Baseline assessment

A baseline assessment of inputs and practices was carried out between December 2010 and August 2011 using three checklists for facility assessment, delivery observation and interview with women after delivery (‘postpartum women’s interview checklist’). The facility assessment checklist covered equipment, supplies and drugs based on standard WHO and Government of India guidelines [29, 30], while the delivery observation and postpartum women’s interview checklists assessed whether essential evidence based care practices were being observed or not. There was some overlap between items on the delivery observation and postpartum women’s interview checklists, so that practices could be assessed from interview whenever delivery observation was not possible. The decision on whether or not a practice was being routinely followed in a facility was based on observation of deliveries and interviews with postpartum women, and identification of the most consistent pattern. For example, if interview with three postpartum women with first deliveries showed that two had an episiotomy, then episiotomy was considered to be a routine practice for that facility. Although most delivery observations occurred in the daytime, interviews with postpartum women allowed us to also assess practices of providers who worked the night shift. Although the bulk of practices were carried out by nurse-midwives, we were not able to measure practices by provider category. A scoring sheet enlisted the 10 and 17 most important inputs and practices respectively, from the standpoint of maternal and perinatal survival or morbidity (Tables 1 and 2). Each practice was given a weighted scored with zero representing incorrect practice or absence of beneficial practice and the higher scores representing correct practice or absence of negative practice. The maximum score for inputs was 12, while that for practices was 25 if a delivery could be observed during the visit, and 19 if it could not be observed. On each visit, the facility was scored and the percentage change of inputs and practices was assessed, with an increasing rate demonstrating implementation of more optimal actions and a decreasing rate demonstrating the opposite. Eight postgraduate social scientists were trained over 4 weeks to carry assessments during facility visits. The state government issued a letter to all participating facilities, positioning the intervention as being one for quality improvement.

**Table 1:** **Scoring sheet for the assessment of practices in labour room and postnatal ward**

Practice indicators	Score	
Practices		
Shaving of pubic hair	Yes = 0	No =1
Enema	Yes = 0	No =1
Partograph chart used	Yes = 1	No = 0
Fetal heart sounds (FHS) heard during labour*	Yes = 1	No = 0
Position of delivery	Lithotomy/Dorsal =0	Sitting =1
Augmentation of labour**	Yes = 0	No =3
Episiotomy for primigravidas	Yes = 0	No =2
Abdominal pressure applied in labour	Yes = 0	No =1
Intramuscular (IM) Oxytocin given after delivery	Yes = 3	No =0
Vaginal packing	Yes = 0	No =1
Proper drying and wrapping of newborn	Yes =1	No =0
Routine suction of all newborn	Yes =0	No =1
Initiation of breast feeding within 1 hour	No =0	Yes =2
Timing of discharge	<12 hours = 0 12–23 hours = 1	>24 hour = 2
Sterile gloves used for delivery	Yes =1	No =0
Hand-washing before conducting delivery	Yes =1	No =0
Postpartum checkup in ward***	Yes =2	No =0
Total practice score for this facility		
Maximum practice score possible		
% score for practices		

*Fetal heart sounds heard during labour: Providers were seen to be listening to fetal heart sounds at least once during period of observation.

**Augmentation of labour: A drug (Intravenous oxytocin/Intramuscular oxytocin/dinoprostone gel/misoprostol) used to augment labour pains.

***Postpartum checkup in the ward: Pulse or BP of mother measured at least once in 12 hours after being shifted to the postpartum ward.

**Table 2:** **Scoring sheet for the assessment of inputs in labour room**

Input indicators		
Ambubag kept ready in LR	Yes = 1	No =0
BP instrument & stethoscope ready in LR	Yes = 1	No =0
Washbasin and running water in LR	Yes = 1	No =0
Autoclaved present in working condition	Yes = 1	No =0
Labour room clean	Yes = 1	No =0
Labour table condition	Clean & has no old blood stuck =1	Dirty & has old blood stuck = 0
Oxytocin available in LR	Yes =1	No =0
Staff in LR SBA trained	All =2	Half =1
Doctors (who conduct delivery) oriented on evidence based care	All =2	Half =1
IEC material (chart ) on evidence based practices displayed in LR	Yes = 1	No = 0
**Input score for this facility**		
**Maximum input score possible**		
% score for inputs		

*Abbreviations*: *LR* Labour room, *SBA* Skilled birth attendant, *IEC* Information education communication.

### The intervention

The intervention comprised of two activities, orientation - training of health staff and visit cycles of regular facility assessment, feedback, training and action.

#### Orientation – training of health staff on evidence based delivery-newborn care and skilled birth attendance

Senior doctors who conducted or oversaw deliveries in the selected facilities, and their district managers were invited to a 1-day orientation workshop on the role of evidence based care in ensuring quality, to discuss barriers and to make plans for improving quality. A total of 25 district managers and 85 doctors participated in the orientation. The guidebook, “Recommendations on key evidence based practices in Rajasthan” was handed out as reference material. Forty senior nurse-midwives posted to labour rooms of these facilities were provided a 21-day training course on skilled birth attendance. Additionally, another 35 nurse-midwives were provided 5 days short intensive training on skilled birth attendance and evidence based delivery care.

#### Bi-monthly/quarterly facility assessment, feedback, training and action (AFTA) visits by a project team

On each scheduled visit, 1–2 project staff went through 4 steps: (a) Assessment and scoring using 3 checklists as for baseline assessment (b) Verbal and written feedback to clinical and managerial staff focusing on shortcomings and ways to overcome them. The team also prepared the facility and district report cards and shared them with block and district health officials, and discussed during bi-annual district and annual state review meetings. (c) Spot orientation–training: Facilitators conducted spot training over 1–2 hours based on specific quality gaps identified during the assessment, using multimedia presentations, guidance materials and bedside examples. The training especially focused on nurses and doctors that had not earlier managed to attend scheduled orientation or training courses. The chart with 19 evidence-based care practices was displayed in labour rooms to act as reminder for providers (d) Action: Visiting facilitators worked with local staff to remedy gaps in equipment and supplies from available stocks and stores, use NRHM discretionary funds to order missing items and/ or re-deploy staff, or to appoint cleaner staff for labour rooms. They also provided feedback to district level officers to facilitate purchase of high value items, recruit or deploy the staff, to nominate a program manager to monitor quality during facility visits, and to facilitate the training of nurse midwives in skilled birth attendance.

### Statistical analysis

Data were entered on a spreadsheet and statistical analyses were performed using Stata (version 11). A significance level of .05 was used throughout the study. McNemar’s exact test was used to determine either the effect of the intervention on practices/inputs at each facility, or across all facilities. Each practice/input was dichotomized (re-coded to 0 or 1, where 1 indicated the presence of a beneficial practice or absence of bad practice, while 0 indicated the opposite. A Wilcoxon-signed rank test was performed in order to evaluate the overall effect that the intervention had on all of the facilities participating in this study.

## Results

After the baseline visit, each of the 44 selected facilities was enrolled in the programme and similar visits were made every 2–3 months for an average of 15 months (range 6 to 20 months). In this way, each facility received 3 to 9 visits (average 5.5). This paper presents findings of the baseline and last visit for each facility, with the latter occurring between March and August 2012.

Characteristics of the participating hospitals during the project period are presented in Table 3. The average annual delivery load of these facilities was 3035 for year 2011.

**Table 3:** **Characteristics of the participating facilities**

	District hospitals (n = 9)	CHCs (n = 32)	PHCs (n = 3)	All
Total annual caseload	67120	62865	3565	133550
Average caseload per facility per year	7458	1965	1188	3035
Caesarean section %	5.2%	0.6%	0	2%
Number of obstetricians posted (average)	3.3	0.8	0	1.3

*Abbreviations*: *CHC* Community Health Centre, *PHC* Primary Health Centre.

Results from the baseline and last visit for the selected outcomes are presented in Tables 4, 5 and 6. We tried to see which practices changed significantly after intervention across all hospitals. Bold font indicates that a particular input/practice changed significantly after the intervention, at the 5% level.

**Table 4:** **Effect of intervention on individual incorrect practices, across all hospitals**

Practice	Baseline	End-line	P value
Shaving of pubic hair	23%	2%	**0.002**
Routine enema	18%	2%	**0.0078**
Dorsal position for delivery	100%	93%	0.25
Augmentation of labour	93%	45%	**0**
Episiotomy for primis	77%	45%	**0.0003**
Fundal pressure	48%	14%	**0.0003**
Vaginal packing	48%	18%	**0.0169**
Routine suction of all newborn	75%	34%	**0.0005**

Bold text reflects the statistically significant differences.

**Table 5:** **Effect of intervention on individual beneficial practices, across all hospitals**

Practice	Baseline	End-line	P value
Partograph chart used	11%	18%	0.4531
FHS heard during labour	9%	48%	**0.0001**
IM oxytocin after delivery	57%	90%	**0.0001**
Proper drying and wrapping of newborn	55%	82%	0.25
Initiation of breast feeding within 1 hour	25%	48%	**0.02414**
Timing of discharge after 24 hours	75%	89%	0.2188
Sterile gloves used for delivery	59%	73%	0.33
Hand-washing before conducting delivery	7%	7%	0.2
Postpartum checkup in ward	11%	5%	0.5

Bold text reflects the statistically significant differences.

**Table 6:** **Effect of intervention on selected inputs, across all hospitals**

Input	Baseline	End-line	P value
Ambubag kept ready in LR	68%	95%	**0.002**
BP instrument & stethoscope ready in LR	45%	75%	**0.009**
Washbasin and running water in LR	80%	98%	**0.004**
Autoclave in working condition	68%	84%	0.1185
Labour room clean	52%	73%	**0.0309**
Labour table clean	52%	59%	0.5034
Oxytocin available in LR	41%	98%	**0**
At least half of nurse midwives in LR SBA trained	80%	91%	0.2188
Doctors oriented on evidence based care	18%	55%	**0.001**
IEC material on evidence based practices displayed in LR	7%	91%	**0**

*Abbreviations*: *LR* Labour room, *SBA* Skilled birth attendant, *IEC* Information education communication.

Bold text reflects the statistically significant differences.

**Figure 2:**
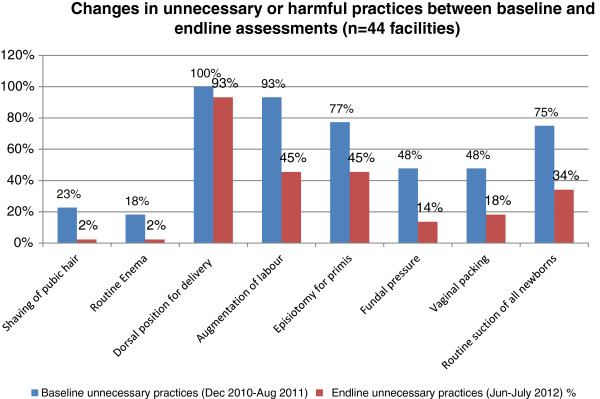
**Changes in unnecessary or harmful practices between baseline and end-line assessments.**

During the baseline period, augmentation of labour was common in 93% of facilities, and episiotomy for primigravidas was a routine in 77% facilities (Table 4 and Figure 2). After the intervention, 48% facilities were providing routine augmentation of labour, and 57% were providing routine episiotomy to primigravidas. Similarly, the proportion of facilities applying fundal pressure reduced from 48% to 14% and those using vaginal packing after delivery reduced from 48% to 18% during the intervention period. Overall, the frequency of almost all incorrect practices reduced in facilities, except for dorsal delivery position. The frequency of routine enema and public shaving was already low in the majority of facilities at baseline, this further reduced over time.

**Figure 3:**
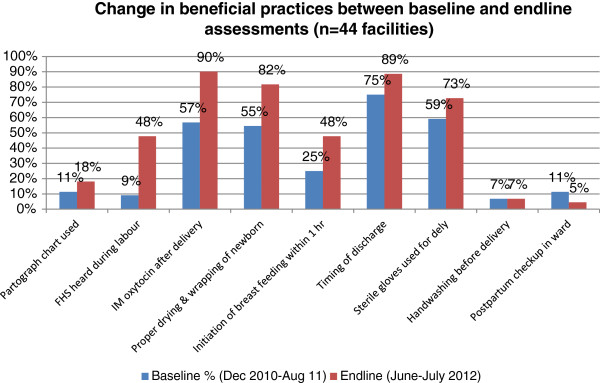
**Changes in beneficial practices between baseline and end-line assessments.**

Among the beneficial and correct practices, the monitoring of foetal heart sounds (FHS) during labour increased from 9% to 48% facilities. Similarly, 57% facilities were using intramuscular (IM) oxytocin during 3^rd^ stage of labour at baseline, this increased to 90%, while initiation of breastfeeding within one hour after birth increased from 25% to 48% facilities (Table 5 and Figure 3). Several other beneficial practices showed marginal improvement, such as drying and wrapping of newborn, timing of discharge after 24 hours, and use of sterile gloves for delivery -- the change was statistically not significant. Similarly, the practice of handwashing before conducting delivery and postpartum checkup of woman and baby in the ward did not show any significant change (Table 5).

**Figure 4:**
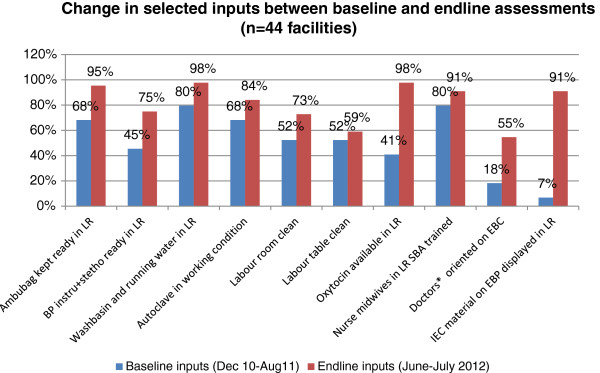
**Changes in selected inputs between baseline and end-line assessments.**

Overall, 10 of the 17 care practices showed significant improvement. In most of the facilities where change occurred, it did so within the first 3–6 months after start of the intervention (data not presented), and then remained stable with some fluctuations. Six out the ten key inputs showed significant improvement -- the availability of an ambu bag, BP instrument and stethoscope oxytocin and a washbasin with running water improved significantly (Table 6 & Figure 4). Similarly, the proportion of facilities with a doctor trained in evidence based care practices and availability of IEC materials improved significantly. However, the proportion of facilities with a clean labour room, clean labour table and an autoclave in working condition did not improve significantly . Similarly, the proportion of facilities in which at least half the nurse midwives were trained in skilled birth attendance was 80% in the beginning, and increased to 91%. This was in part because of rotation of nurse-midwives’ duties to wards and locations outside the labour room.

### Changes in practices at each individual facility

**Figure 5:**
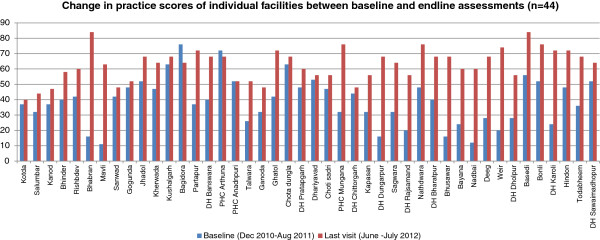
**Changes in practice scores of individual facilities between baseline and end-line assessments.**

On comparing the baseline and end line scores for care practices in individual facilities, we found that scores improved for except three (Figure 5). The average baseline score was 39% (range 11 to 76%), which increased to 63% after the intervention (range 40-84%). The average increase in practice score was 24% (range 0-68%), however, there were variations across facilities.

## Discussion

The results of this intervention show that a system of regular visits to facilities, based on a cycle of assessment, feedback, training and action at public health facilities, backed by policy makers can lead to substantial improvement in adherence to evidence based care practices. Quality of intrapartum and immediate postpartum care is closely linked to better maternal and perinatal outcomes, and our results suggest a potential approach for improving the quality of childbirth services.

Of 17 selected practices, significant improvements were seen in 10 practices. Most importantly, several unnecessary practices reduced significantly, such as augmentation of labour, routine episiotomy and vaginal packing. Similarly, the use of several beneficial practices increased, such as monitoring of foetal heart sounds during labour, use of oxytocin in 3rd stage of labour, and initiation of breastfeeding within one hour after birth.

In terms of perinatal outcomes, the single most significant change was reduction in the rates of routine augmentation of labour, which was practiced in 93% facilities at baseline, and reduced to 45% facilities after the intervention. Convincing providers to avoid routine augmentation of labour was difficult to achieve, since most preferred quick delivery, which reduces the need for monitoring of labour. Most providers also preferred to complete a delivery before their shift change, encouraging them to expedite the delivery process.

There was a significant reduction in the proportion of facilities practicing routine episiotomy for primigravidas, however this change was also challenging to achieve. Providers did not readily give up the practice of routine episiotomy; some doctors vehemently argued with members of the visiting team saying “are you trying to make us *dais* (traditional birth attendants)?”. Some doctors dismissed all the assembled evidence and said that they would rather go by what they have learnt from their seniors in medical colleges.

Some practices did not show significant improvement -- these included avoidance of lithotomy position for delivery, use of a partograph, hand-washing, use of sterile gloves for delivery, and postpartum check in the ward. Based on our interactions with staff during feedback and training sessions, we surmised that the reasons for not changing a practice were linked to not being fully convinced of the value of changed practice, lack of supplies, fear of being ridiculed by colleagues, or that the practices meant greater time and effort for providers. For example, some providers were convinced about the need to deliver women in an upright position and tried it out only during night duties when other staff was not present and watching. The lack of postpartum monitoring might be related to the fact that women and newborns were routinely discharged 48 hours after delivery without an assessment of the maternal or neonatal condition. Hence introduction of discharge criteria could encourage post partum checks to ensure fitness for discharge. Further, it would be critical to have medical colleges strictly follow and advocate evidence based care practices.Greater monitoring of quality by district level authorities would help to bring about changes in practices such as postpartum monitoring and handwashing.

In our view, the underlying factors through which this intervention improved childbirth practices were: (1) providers understanding the rationale for the necessity of a change and its benefits for maternal or perinatal outcomes, which occurred through orientation of doctors and discussions during repeated facility visits. We found that most changes occurred when senior doctors or senior labour room nurse-midwives took on an active role in encouraging behavior change in their facilities. (2) repeated visits by program staff along with educational materials acted as reminders, (3) endorsement by persons in authority either through office orders or during review meetings, (4) the new practice reduced the workload of staff (for example, avoiding the routine enema or pubic shaving), (5) facilitating the availability of supplies or equipment by program staff, and (6) the on-site training on specific issues was useful to convince providers of the need for change in practices, especially for nurse midwives. Orientation in small groups allowed local clinical staff to openly express their concerns and questions, and discuss issues specific to their facility. One of the most powerful aspects of on-site training was the link between moral and ethical responsibility and adverse outcomes, if evidence based practices are not followed.

Simultaneously, there were some barriers that prevented the improvement of clinical practices: (1) medical colleges as role models – many doctors challenged the evidence, stating that they would do what they had learnt and observed in their medical colleges, (2) the new practice meant greater effort for providers, for example, the monitoring of fetal heart sounds during labour or postpartum checkup would mean more work for providers, and (3) lack of skills or confidence in performing a new practice, for example, many providers expressed that they were not able to conduct a delivery in upright position. A qualitative study in Latin American hospitals has identified barriers to change at level of individual providers, hospitals policies and macro level factors; and suggests that interventions must attempt to bring about sustainable change at all levels [31]. Other studies have also identified that the common barriers to quality improvement are leaders’ and clinicians’ knowledge, attitudes, and practices, and the implementation climate [28]. Medical colleges and professional associations in India have not paid sufficient attention to evidence based childbirth care thus far, and at one time even promoted “programmed labour” which includes routine augmentation [32].

While poor quality of childbirth services has been recognized as a major barrier for reduction of maternal and perinatal mortality in different settings [32, 33], successful experiences of improving quality are limited. Many approaches limit themselves to assessing quality without intervening to improve quality. Our intervention attempted to make an impact both at individual and system level. Examples of actions at the individual level were guidance materials, orientation and training programs and feedback to providers, while examples of actions at the system level were feedback to district health officials, who in turn took necessary steps to address the gap. Further, repeated visits to the facility were crucial in maintaining the changes.

Experience from other settings has demonstrated the effectiveness of various approaches to bring about change in evidence based care practices. In an intervention study in Ukraine, training of staff on evidence based guidelines resulted in significant decrease in harmful practices and adherence to protocols [34], mostly by first 3 months of intervention followed by its being sustained. In a study from Turkey, practices to speed up labour (early amniotomy and augmentation with oxytocin) were identified as very common and were linked to financial incentives for a quick delivery [35]. In our study, the social pressure to comply with practices recommended by supervisors and peers played a major role in adopting evidence based procedures. In a pilot study in a single hospital in Karnataka, India, use of a childbirth quality checklist by providers led to significant improvement in quality of care [36]. However, scaling up the approach in greater number of hospitals, coupled with a longer follow-up will demonstrate the effectiveness of this approach. Another study in an Iranian hospital involved building a professional consensus to identify the priority evidence based recommendations and designing new model of care by involving the physicians in the hospital also showed promising results [37].

An overview of systematic reviews of strategies [38] to improve quality of maternal and child care has shown that no single strategy is effective in changing professional practice, and that multiple approaches should be combined to improve quality. The reviewers also conclude that organizational interventions might be important, given the wide prevalence of underlying organizational or system problems.

### Limitations

Parijaat was designed as a health system intervention and not as a study, hence there are certain limitations to the data and its interpretation. The government supported intervention did not allow for control facilities to enable comparison. The intervention started at different times in different facilities resulting in somewhat different periods of intervention and visits across facilities. An important tool for assessing the practices was observation of labour and delivery, which depended on whether a woman was in labour at the time of scheduled facility visits. Hence some assessments were based on interviews with women that had delivered on the previous day or two. It is possible that some providers improved their practices in presence of facilitators, which could affect the results of delivery observation. However, to avoid this, facilitators combined the results of observations with those from interviews with postpartum women, which in turn would reflect practices in absence of a facilitator. Although we have only compared data from the first and last visits, we observed that most change was established and sustained within 6 months of initiating the intervention. Hence we feel confident about attributing change to the intervention.

### Strengths

Among the strengths of this intervention is the development of assessment tools based on a review (from literature and experience) of childbirth practices in Rajasthan and by building consensus among local stakeholders. Secondly, the intervention was implemented across large numbers of institutions in 10 districts which represented a diversity of settings, hence the feasibility of scaling up is high. These selected institutions conducted the majority of deliveries in their area, hence the intervention has a potential to make an impact on a larger proportion of deliveries. Further, the intervention attempted to make the quality improvement part of the organizational culture by building a professional consensus and providing regular feedback to providers, managers and administrators.

A question that remains is whether changes will last if the system of monitoring visits by project staff were to be withdrawn. Although project teams were accompanied by government staff on about 25% visits, we feel that while practices are likely to be sustained where providers were convinced about the value of change and found it convenient to do so. Reviews of sustainability suggest that quality improvement in health services are likely to sustain if they become part of organizational culture [39, 40]. Other experiences that show that quality improvement efforts need to be repetitive even after reaching a mature phase, and that efforts have to be focussed at all levels from frontline providers to senior leaders within the organization and also involve external structures such as national quality control boards or regulators [41]. It has been shown that continuously monitoring quality indicators significantly improved the quality of care [42, 43]. In a qualitative study of policy makers and program managers in India, poor monitoring of health systems has been identified as an important bottleneck to improving quality of delivery services [44]. Hence we feel that the quality improvement efforts should continue in order to sustain the change, and should be institutionalised through state level regulatory bodies.

## Conclusions

The results of this intervention demonstrate that substantial improvement in childbirth practices can be achieved through regular visits to facilities for quality assessment and improvement, along with orientation training of service providers, and provision of guidance materials. Close collaboration with state and district level decision makers is crucial to build consensus and to sustain the quality improvement process. Our results also show that this approach can be delivered at scale, and could act as a model for other similar settings witnessing serious deficiencies in quality of intrapartum care. Further changes in practices would require advocacy with medical colleges and professional bodies.
